# GLP-1R biased cAMP agonism maintains glycemic control with reduced malaise and emesis in preclinical mammalian models

**DOI:** 10.1111/dom.70427

**Published:** 2026-01-08

**Authors:** Caitlin Baumer-Harrison, Danya Aldaghma, Alex D. White, Sarah V. Applebey, Allison M. Pataro, Allaha Z. Mohiby, Brandon Alonso, Allison G. Xiao, Libbey S. O’Farrell, Yuewei Qian, Tamer Coskun, Matthew P. Coghlan, Francis S. Willard, Minrong Ai, Kyle W. Sloop, Robert P. Doyle, Tito Borner, Bart C. De Jonghe, Matthew R. Hayes

**Affiliations:** 1 Department of Psychiatry, Perelman School of Medicine, University of Pennsylvania, Philadelphia, Pennsylvania, USA; 2 Department of Biobehavioral Health Sciences, School of Nursing, University of Pennsylvania, Philadelphia, Pennsylvania, USA; 3 Division of Diabetes and Complications, Lilly Research Laboratories, Lilly Corporate Center, Indianapolis, Indiana, USA; 4 Department of Biological Sciences, University of Southern California, Los Angeles, California, USA; 5 Department of Chemistry, Syracuse University, Syracuse, New York, USA; 6 Department of Medicine and Pharmacology, State University of New York, Upstate Medical University, Syracuse, New York, USA

**Keywords:** *β*-arrestin, biased agonist, emesis, exendin-4, GLP-1R, malaise, nausea

## Abstract

**Aims:**

Glucagon-like peptide-1 receptor (GLP-1R) agonists improve glycemic control and promote weight loss in diabetes and obesity but are also associated with gastrointestinal adverse events, including nausea and emesis in many patients. These concerns highlight the need for the development of novel GLP-1R agonists that minimize these side effects while maintaining beneficial metabolic outcomes. Here, we investigate the *in vivo* effects of exendin-4-Phe1 (Ex-Phe1), a GLP-1R biased agonist.

**Materials and Methods:**

In three pre-clinical species, mice (*n* = 43), rats (*n* = 54), and musk shrews (*n* = 30), we examined *in vivo* glycemic control, feeding, and nausea/emesis following native Ex-4 and Ex-Phe1 administration. We also used cFos expression following Ex-4 and Ex-Phe1 administration to examine neural activation in regions involved in mediating nausea and emetic side effects of GLP-1R agonism.

**Results:**

*In vitro* studies show Ex-Phe1 favors cAMP signaling with reduced β-arrestin recruitment. Compared to Ex-4, Ex-Phe1 produced fewer emetic episodes in musk shrews (*Suncus murinus*) and little to no pica, a proxy for nausea, in rats. Ex-Phe1 effects on food intake and body weight varied by species, while Ex-4 and Ex-Phe1 similarly enhanced glucose tolerance in all species. Ex-4 and Ex-Phe1 increased cFos expression within brain regions linked to nausea and emesis in all species.

**Conclusions:**

Collectively, Ex-Phe1 maintains glycemic benefits in all three species, but putatively blunts the ability of the CNS GLP-1R+ cells to drive anorexia and weight loss, as well as unwanted adverse events (nausea/emesis) in rats and musk shrews.

## 1 | INTRODUCTION

Glucagon-like peptide-1 (GLP-1R) agonists are widely used in the United States for the treatment of type 2 diabetes (T2DM) and obesity as they enhance postprandial insulin secretion and reduce food intake and body weight.^1–3^ Unfortunately, the use of these drugs is also associated with adverse effects. The most reported side effects associated with GLP-1R agonists are gastrointestinal symptoms, including nausea, vomiting, and slowing of gastric emptying.^4–6^ Therefore, there is a need to develop novel GLP-1R agonists that have a reduced incidence of adverse side effects.

The GLP-1R is a G-protein coupled receptor (GPCR); thus, its activation leads to coupling with intracellular effectors, promoting cAMP production and *β*-arrestin recruitment, facilitating receptor internalisation.^6^ These characteristics of GLP-1Rs provide the opportunity to specifically target, pharmacologically, the balance between cAMP production and receptor internalisation to drive desired effects, a process known as biased agonism.^7^ Modulation of GPCR signalling through biased agonism offers the potential to understand the differential effects of receptor activation for enhanced therapeutic outcomes with reduced side effects. Biased agonism of the μ-opioid^8^ receptor, in which G-protein signalling is favoured with limited *β*-arrestin recruitment, is associated with fewer side effects, including nausea and vomiting, and stronger analgesic effects.^9–11^ Exendin-4-Phenylalanine1 (Ex-Phe1), a modified version of the GLP-1R agonist exendin-4 (Ex-4), induces biased agonism of the GLP-1R resulting in similar cAMP signalling and reduced *β*-arrestin recruitment relative to native Ex-4.^12^ While the in vitro effects of Ex-Phe1 are clear, the physiological and behavioural effects are less understood. Studies in fasted mice maintained on a high-fat/high-sugar diet (HFHS) reveal administration of Ex-Phe1 improves glycemic control and acutely suppresses food intake similarly to Ex-4.^12^ However, chronic administration of Ex-Phe1 in lean mice produced no significant effect on food intake or body weight compared to Ex-4, but did improve glycemic control.^12^ These findings indicate dosing and frequency of treatment must be considered in presuming the broad physiological effects of Ex-Phe1.

Current studies are designed to determine if biased agonism of the GLP-1R provides a potential novel approach to GLP-1R based therapeutics. However, little is known about the physiological effects of biased agonism in pre-clinical models, particularly if Ex-Phe1 mitigates nausea and emesis associated with GLP-1R agonism. While previous studies in mice^12^ demonstrate reduced pica behaviour, measured by kaolin intake, compared to Ex-4, mice are not an ideal model for assessing such behaviour. Thus, to understand the effects of Ex-Phe1 on nausea and emesis, studies must be performed in animal models that demonstrate pica behaviour, such as rats, or are capable of emesis, such as musk shrews (*Suncus murinus*). To this end, we report in vitro and biobehavioral analyses comprehensively comparing acute or chronic administration of Ex-Phe1 and Ex-4 in emetic (musk shrews) and non-emetic preclinical models (mice and rats).

## 2 | METHODS

### 2.1 | Experimental models

All animals were acclimated to single housing in their home cage and habituated to injections for at least 1 week prior to experiment onset. Animals were naive to experimental treatments and tests prior to the start of the experiment. All procedures were approved by the Institutional Care and use Committee of the University of Pennsylvania and Eli Lilly and Company.

Adult male mice on a C57BL/6J background (Taconic) weighing ~48–53 grams (*n* = 43) at arrival were housed in plastic cages. Adult male Sprague-Dawley rats (Charles River) weighing ~250–275 g (*n* = 54) at arrival were housed in hanging wire-bottomed cages. Adult male and female shrews (*S. murinus*; University of Pennsylvania) weighing ~50–70 g (*n* = 20) and ~30–50 g (*n* = 9), respectively, were housed in plastic cages (37.3 × 23.4 × 14 cm, Innovive). All animals were maintained on a 12:12 h light/dark cycle in temperature- and humidity-controlled rooms. Tap water was available ad libitum unless stated otherwise. Mice were given high-fat, high-sugar diet (40% fat, 20% sugar; Teklad Diet, catalog TD95217). Rats were given high-fat diet (60 kcal% fat; Research Diets, D12492), and kaolin pellets (Research Diets, K50001). Shrews were given feline food (27.364% fat, Laboratory Feline Diet 5003, Lab Diet) unless stated otherwise. All diets and kaolin were available ad libitum unless stated otherwise.

### 2.2 | Method details

#### 2.2.1 | Drugs

Two drugs were used for these studies: exendin-4 (Ex-4, MW = 4187 g/mol; Tocris Biosciences) and phenylalanine1-Ex-4 (Ex-Phe1, MW = 4196 g/mol; Eli Lilly and Company or Wuxi). For studies in mice, drugs were dissolved in 40 mM Tris and delivered by a subcutaneous osmotic mini pump (Alzet) for 11 days. For studies in rats, drugs were dissolved in saline and injected intraperitoneally (IP) at a volume of 1 mL/kg or delivered by a subcutaneous osmotic mini pump (Alzet) for 12 days. For studies in shrews, drugs were dissolved in saline and were injected IP at a volume of 1 mL/kg.

#### 2.2.2 | Dose comparison of Ex-Phe1 and Ex-4 on energy balance in mice

20-week-old male mice fed with high fat high sugar (HFHS) diet for 14 weeks were surgically implanted with Alzet pump subcutaneously for continuous delivery of Ex-4 (12.6 and 126 μg/kg/day), Ex-Phe1 (12.6 and 126 μg/kg/day) or vehicle (40 mM Tris, pH 8). Doses were selected based on the EC50 for Ex-Phe1 and drugs were administered via mini-pump to compare to studies that tested a lower dose of Ex-Phe1.^12^ Body weight and food intake were monitored daily for 11 days. Quantitative nuclear magnetic resonance was performed at day 1 and day 11 of the study.

#### 2.2.3 | Dose comparison of Ex-Phe1 and Ex-4 on energy balance and malaise in rats

To compare the effects of Ex-Phe1 and Ex-4 on energy balance, rats (*n* = 30) were chronically administered Ex-4 (3 μg/kg), Ex-Phe1 (3 μg/kg), or vehicle (1 mL/kg sterile saline) two times a day for 14 days. This dosing regimen is consistent with previous studies that show significant kaolin intake, food intake, and body weight suppression following chronic Ex-4 administration (3 μg/kg, b.i.d.).^13^ High-fat (HF) diet intake and body weight were measured daily for the duration of the study. Kaolin intake (an established model of nausea in rats^14,15^) was also measured daily for the duration of the study. All intake measurements accounted for food spillage.

For continuous, subcutaneously-delivered Ex-Phe1 and Ex-4, rats (*n* = 21) were exposed to kaolin and HF diet intake for 12 weeks. They were assigned to one of three weight-matched treatment groups (0.9% saline vehicle, 7 μg/kg/day Ex-Phe1, or 7 μg/kg/day Ex-4). On day 0, the rats were implanted with prefilled, primed osmotic minipumps (Alzet model 2ML4) and thereafter body weight, food intake, and kaolin intake were measured every 48 h for 12 days. Please see the Supplementary Appendix for additional details.

#### 2.2.4 | Dose comparison of Ex-Phe1 and Ex-4 on energy balance and emesis in shrews

In this study, shrews (*S. murinus*) were used as a model to test the effect of the biased agonist Ex-Phe1 in comparison to Ex-4 on food intake and body weight. The advantage of using musk shrews to study nausea/emesis is that they are a small mammalian model capable of vomiting, unlike rodents, with a large body of literature on GLP-1 and feeding and emetic behaviour.^16–20^ However, they do require higher dosing compared to mice and rats due to their high metabolic rate. Prior to the experiment, male shrews (*n* = 10) were acclimated to emesis boxes, transparent plastic observation chambers (dimensions: 23.5 × 15.25 × 17.8 cm), and habituated to IP injections for two consecutive days. Two hours before dark cycle onset, animals were injected with Ex-4, Ex-Phe1, or vehicle, and a video was recorded using a Vixia HF-R62 Canon camera for 120 min. After that, animals were returned to their cages. A blinded observer analysed the occurrence and frequency of emetic episodes, defined by intense rhythmic abdominal contractions, which could involve either oral expulsion (vomiting) or contractions without material expulsion (retching). The total number of episodes was measured.

For food intake measurements, a custom-made feedometer system developed by the De Jonghe laboratory as described,^16^ was employed.^19,21^ After returning the animals from the emesis boxes to the feedometer cages, food intake was manually measured at 6, 24, and 48 h after drug administration. Body weight was also measured at 0, 24, and 48 h. Each treatment was separated by 72 h.

#### 2.2.5 | Dose comparison of Ex-Phe1 and Ex-4 on glycemic control in rats

Three hours before dark phase onset, chronically treated rats (*n* = 30) and acutely treated rats (*n* = 15) were deprived of food and water prior to testing. Blood glucose (BG) was measured from a small drop of blood collected from the tip of the tail and analysed using a standard glucometer (AccuCheck). Immediately after baseline BG measurements (*t* = −30 min), rats received an IP injection of Ex-4 (3 μg/kg), Ex-Phe1 (3 μg/kg), or vehicle (1 mL/kg sterile saline). 30 min after drug injection (*t* = 0 min), BG was measured and each rat was administered glucose (2 g/kg) via oral gavage. BG measurements were taken at 20, 40, 60, and 120 min following glucose gavage. Food and water were returned after the final measurement.

#### 2.2.6 | Dose comparison of Ex-Phe1 and Ex-4 on glycemic control in shrews

Three hours before the onset of the dark phase, female shrews (*n* = 9) were deprived of food and water. At *t* = −30 min, BG glucose levels were measured using a standard glucometer (AccuCheck) from a small blood sample taken from the tail. Immediately after that, each shrew received an IP injection of different doses of Ex-4, Ex4-Phe, or vehicle (sterile saline, 1 mL/100 g body weight). BG levels were measured again 30 min later (*t* = 0 min), followed by an IP bolus of glucose (2 g/kg). Right after the glucose injection, subsequent BG readings were taken at 20, 40, 60, and 120 min. Water was returned after the first drug injection (*t* = −30 min), and food was restored after the final measurement.

#### 2.2.7 | In vitro assessment of cAMP accumulation and *β*-arrestin recruitment

##### Proteins

Protein sequences used in this study are as follows: human GLP1R: NP_002053.3; rat GLP1R: P32301.1; mouse GLP1R: NP_067307.2; Etruscan shrew GLP1R: XP_049621334.1; human ARRB1: NP_004032.2; rat ARRB1: NP_037042.1; mouse ARRB1: NP_796205.1; Etruscan shrew ARRB1: based on XP_049636255.1, XP_049636256.1, XP_049636257.1, and homologous sequence analysis of human, rat and mouse GLP1R; human ARRB2: NP_004304.1; rat ARRB1: NP_037042.1; mouse ARRB2: NP_663404.1; Etruscan shrew ARRB2: XP_049630962.1. The nucleotide sequences encoding various GLP1R followed by GS linker (GGGGS) and HaloTag (Promega) were inserted into pcDNA3.1 (Invitrogen), and the nucleotide sequences encoding NanoLuc luciferase (NLuc, Promega) followed by GS linker and various ARRB1 or ARRB2 were inserted into pcDNA3.1hyg (Invitrogen). All expression constructs were verified by DNA sequencing.

##### cAMP accumulation assay

HEK Freestyle cells (ThermoFisher Scientific, R79007) seeded in suspension at a density of 250 000 cells/mL were transiently transfected as previously described^22^ with Halo-tagged GLP1R constructs using Fugene-6 (Promega, E2691). After 48 h, transfected cells were spun down and resuspended in cAMP assay buffer (DMEM, Gibco 31053) containing 0.1% (w/v) casein (Sigma, C4765). Cells were then added in suspension at a density of 1000 cells/well to 384-well white microplates (Costar, 3570) containing a range of ligand concentrations that were prepared via acoustic direct dilution in assay buffer containing 250 μM IBMX (total reaction volume of 20 μL), followed by incubation for 30 min at 37°C. Cells were then lysed via sequential addition of 10 μL d2-labelled cAMP competitor conjugate and 10 μL cryptate-conjugated detection antibody (Revity, 62AM4PEC), then incubated at room temperature for 1 h followed by quantification of time-resolved fluorescence resonance energy transfer using an Envision platereader with calibration of ratios to external synthetic cAMP standards contained in a plate that was processed in parallel. Normalised percent values were fit to the four-parameter logistic model using Graphpad Prism.^23^

##### β-*arrestin recruitment assay*

*β*-arrestin (*β*arr) coupling to the GLP-1R was assessed via BRET in HEK Freestyle cells using the NanoBRET detection system (Promega, N1663). Briefly, cells were seeded in suspension at a density of 250 000 cells/mL and transfected with species-specific pairs of GLP1R-Halotag and either *β*arr-1 or *β*arr-2N-terminally fused to NanoLuc using Fugene-6. After 24 h, cells were reseeded in suspension at a density of 200 000 cells/mL and incubated with either Halo ligand or DMSO overnight. The next day, cells were spun down and resuspended in assay buffer (DMEM, Gibco 30153) containing 0.1% (w/v) casein and luciferase substrate. Cells were then transferred to 96-well white microplates (Corning, 3917) containing a range of agonist concentrations that were prepared via acoustic direct dilution as was done for the aforementioned cAMP accumulation experiments (total reaction volume of 100 μL). After 30 min of incubation at room temperature, emission intensities were measured at 460 nm for donor and 610 nm for the acceptor using an EnVision plate reader. Data analysis was performed using GraphPad Prism software.

#### 2.2.8 | Assessment of neuronal activation in the DVC following Ex-Phe1, Ex-4, or saline treatment

8-week-old male BL6 mice, weighing ~20–25 g, were used for a c-Fos study (*N* = 6 per group). Ex-4 and Ex-Phe1 were dosed subcutaneously at 84 μg/kg. Mice were induced and maintained by continuous isoflurane anaesthesia 1 h post drug treatment. Animals were perfused with cold PBS for 2 min at 20–25 mL/min rate. Brains were then dissected, placed on crushed dry ice and stored in −70°C freezer. Brains were sectioned at 8 μm using a Leica CM3050 S cryostat (Leica Mircosystems) and collected onto SuperFrost Plus microscope slides. Sections were stored at −70°C. RNA in situ (ISH) of c-Fos gene was performed using the RNAscope^™^ Multiplex Fluorescent V2 Assay (Advanced Cell Diagnostics, Cat. 323100). Quantification was performed by VisioPharm software at area of interest: AP and NTS.

Body weight-matched rats (*n* = 30) were used to assess Fos expression within the AP/NTS following IP injection of Ex-4 (3 μg/kg, *n* = 10), Ex-Phe1 (3 μg/kg, *n* = 10), or vehicle (*n* = 10). Food and water were removed 2 h before injections. Two hours following injections, rats were deeply anaesthetised by IP injection of ketamine, xylazine, and acepromazine (180, 5.4, and 1.28 mg/kg, respectively) and transcardially perfused with 0.1 M PBS followed by 4% PFA (in 0.1 M PBS, pH 7.4, ChemCruz). Brains were removed and post-fixed in 4% PFA for 48 h and then stored in 30% sucrose at 4°C until further processing. Brains were sectioned at 30 μm using a Leica CM3050 S cryostat (Leica Mircosystems), collected, and stored in cryoprotectant (30% sucrose, 30% ethylene glycol, 1% polyvinylpyrrolidone-40, in 0.1 M PBS) at −20°C until further processing.

IHC was used to evaluate Fos expression in the AP/NTS after Ex-4, Ex-Phe1, and vehicle treatment and was performed on DVC sections in wells. All steps were performed on a shaker at room temperature unless stated otherwise. Sections were rinsed in 0.1 M PBS and blocked in blocking solution (5% normal donkey serum, 0.3% Triton X-100, 1X PBS) for 1 h. Sections were then incubated overnight with rabbit anti-Fos antibody (1:1000 in blocking solution; s2250; Cell Signalling) at 4°C. Sections were then rinsed in 0.1 M PBS to remove the primary antibody and incubated in secondary antibody donkey anti-rabbit Alex Fluor 488 (1:500 in blocking solution; Jackson Immuno Research Laboratories) for 2 h. After the final rinses in 0.1 M PBS sections were immediately mounted to slides and coverslipped with antifade mounting medium with DAPI (Vectashield). DVC neurons expressing c-*fos* were visualised and images captured at 10× or 20× magnification with a Keyence fluorescence microscope BZ-X800 using Keyence BZ-X800 viewer software. Images were analysed and Fos positive neurons quantified using ImageJ. Fos expression was quantified by counting the number of Fos-positive nuclei per section within the AP and NTS. To determine the mean number of Fos-positive nuclei per section, values across sections were averaged for each animal and then the group mean was calculated.

Body weight-matched shrews (*n* = 11) were also used to evaluate Fos expression in the AP/NTS after IP injection of Ex-4 (210 μg/kg, n = 3), Ex-Phe1 (210 μg/kg, *n* = 4), and vehicle (*n* = 3) treatment using the same procedure as described in rats.

### 2.3 | Statistical analysis

All data are expressed as mean ± SEM and *p* <0.05 was considered significant for all statistical tests. Biobehavioral parameters were analysed using ordinary or repeated measures one-way or two-way ANOVA followed by Tukey’s post hoc test. BG and areas under the curve were analysed using ordinary or repeated measures one-way or two-way ANOVA followed by Tukey’s post hoc test. Analysis of c-Fos and Fos quantification was conducted using unpaired t tests. All statistical analyses were performed with Prism GraphPad Software.

## 3 | RESULTS

### 3.1 | Pan-species in vitro pharmacology of GLP-1R ligands

**FIGURE 1:**
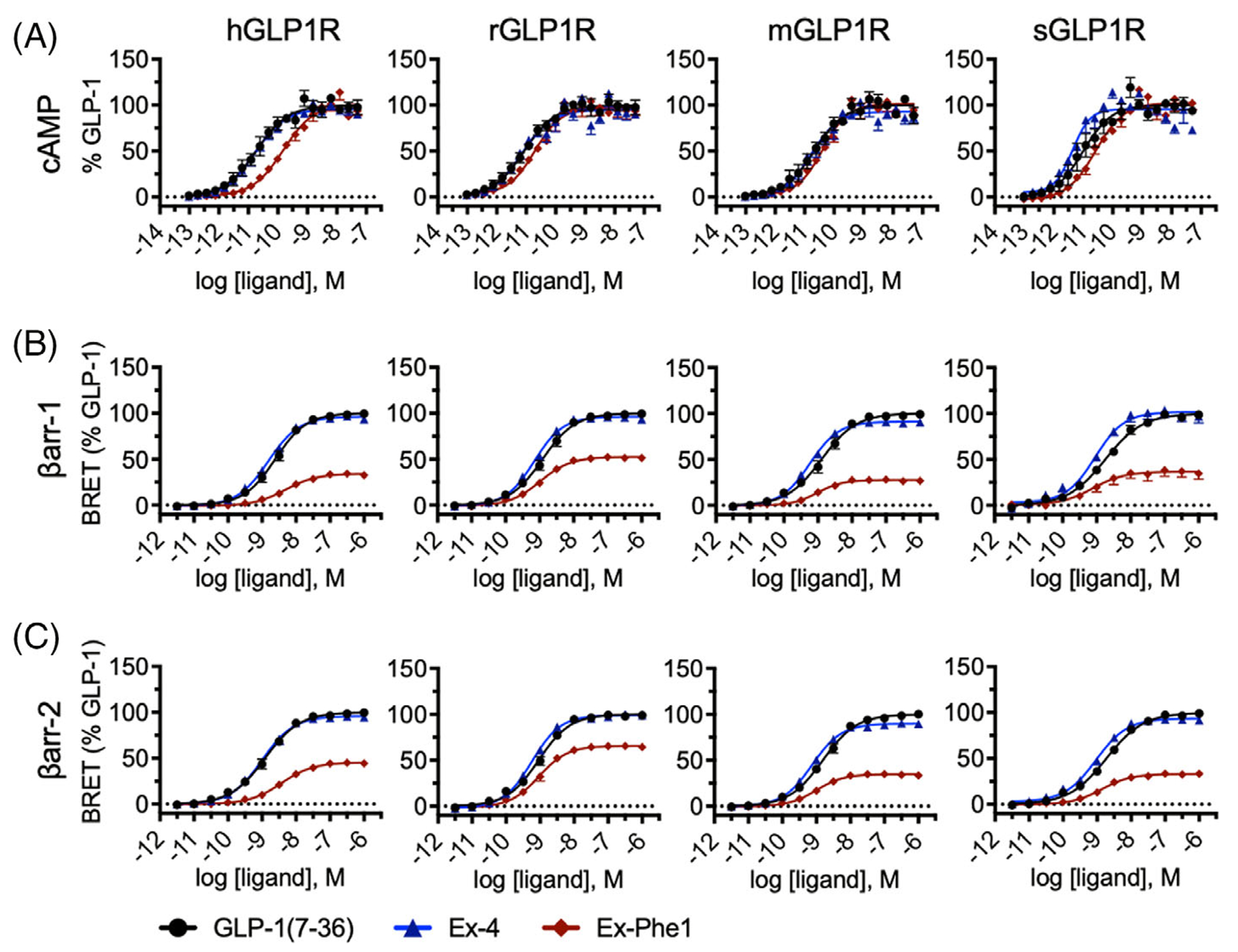
cAMP accumulation and *β*arr recruitment to different species of GLP1R. (A) Concentration-response curves for cAMP accumulation in response to either GLP-1 (7–36), Ex-4, or Ex-Phe1 using HEK Freestyle cells transiently expressing the indicated species-specific GLP1R construct. Data represent the mean values ± SEM of *N* = 3 independent experiments performed with 5 replicates at each point. (B and C) Concentration-response curves for the formation of (B) GLP1R-*β*arr1 or (C) GLP1R-*β*arr2 complexes in response to either GLP-1 (7–36), Ex-4, or Ex-Phe1 using HEK Freestyle cells transiently expressing the indicated species-specific GLP1R-*β*arr construct pairs. Data represent the mean values ± SEM of *N* = 3 independent experiments performed with 4 replicates at each point.

To better understand the pharmacology of biased and unbiased GLP-1 agonists in preclinical animal models, we undertook a multi-species analysis of GLP-1R pharmacology. We used cells independently expressing human, mouse (*Mus musculus*), rat (*Rattus norvegicus*), and shrew (*Suncus etruscus*) GLP-1R and quantified GLP-1 (7–36), Ex-4, and Ex-Phe1 induced cAMP accumulation and *β*-arr recruitment (Figure 1, and Supplemental Table 1). Consistent with the literature, GLP-1 (7–36) and Ex-4 are potent full agonists for cAMP accumulation and *β*-arr1 and *β*-arr2 recruitment, whereas Ex-Phe1 exhibited a characteristic low efficacy for arrestin recruitment consistent with reports of biased agonist pharmacology.^12^ Quantification of the ligand pharmacology at the 4 species (human, mouse, rat, and Etruscan shrew [*S. etruscus*]) indicated that there were no substantial differences in ligand potency or efficacy between species (Supplemental Table 1). Thus, in vivo pharmacological conclusions about target engagement can be generalised across the model systems.

### 3.2 | Effects of chronic GLP-1R agonism versus biased agonism in mice

**FIGURE 2:**
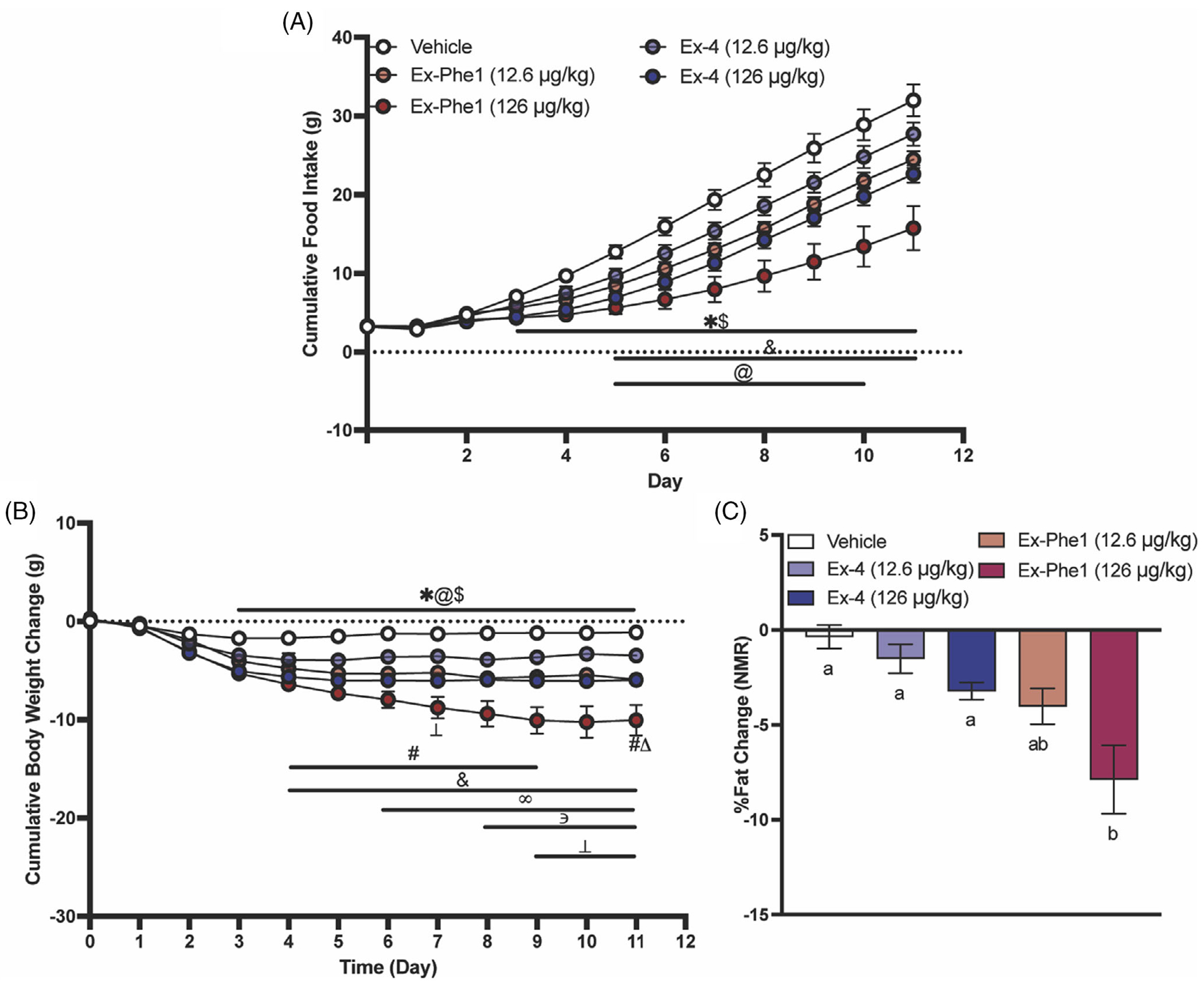
Effects of chronic treatment with Ex-Phe1 in HFHS mice. (A) Ex-Phe1 suppressed HFHS diet intake more robustly than Ex-4 at similar doses (*n* = 5/group). (B) Ex-Phe1 reduced body weight to a greater magnitude than Ex-4 at similar doses. (C) Treatment with Ex-Phe1 (126 μg/kg) induced significant fat loss measured by NMR (*n* = 5/group). All data are expressed as mean ± SEM. Data in (A, B) were analysed with two-way repeated-measures ANOVA followed by Tukey’s post hoc test (*p* <0.05): * Vehicle versus Ex-4 (126 μg/kg), @Vehicle versus Ex-Phe1 (12.6 μg/kg), $ Vehicle versus Ex-Phe1 (126 μg/kg), # Vehicle versus Ex-4 (12.6 μg/kg), and Ex-4 (12.6 μg/kg) versus Ex-Phe1 (126 μg/kg), ∞ Ex-Phe1 (12.6 μg/kg) versus Ex-Phe1 (126 μg/kg), ∋ Ex-4 (126 μg/kg) versus Ex-Phe1 (126 μg/kg), ⊥ Ex-4 (12.6 μg/kg) versus Ex-4 (126 μg/kg), Δ Ex-4 (12.6 μg/kg) versus Ex-Phe1 (12.6 μg/kg). Data in (C) were analysed with one-way ANOVA followed by Tukey’s post hoc test. Means with different letters are significantly different from each other (*p* <0.05).

To further understand the effects of Ex-Phe1 in mice, daily food intake and body weight measurements were obtained from mice maintained on HFHS diet and chronically administered Ex-Phe1 (12.6 or 126 μg/kg), Ex-4 (12.6 or 126 μg/kg), or vehicle by a subcutaneous osmotic mini pump for 11 days. As shown in Figure 2, we observed a dose-dependent decrease in food intake and body weight by either Ex-4 or Ex-Phe1 treatment. Interestingly, Ex-Phe1 treatment at 126 μg/kg/day led to significantly more body weight loss and food intake reduction compared to Ex-4 (126 μg/kg/day) treatment. Ex-Phe1 treatment also led to more fat mass reduction compared to Ex-4 treatment (Figure 2C).

### 3.3 | Effects of chronic GLP-1R agonism versus biased agonism in rats

**FIGURE 3:**
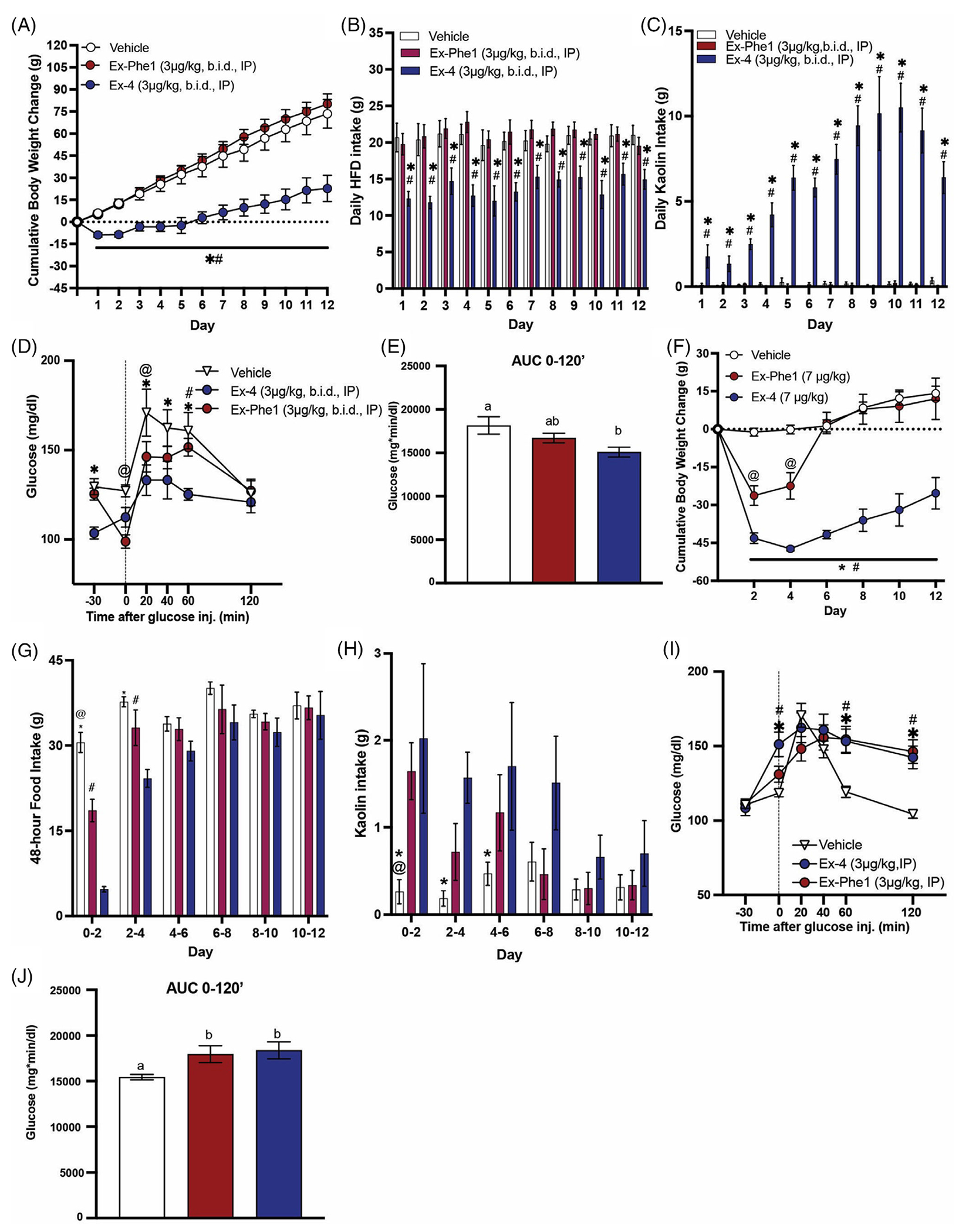
Effects of chronic treatment with Ex-Phe1 in HFD rats. (A) Ex-Phe1 chronic administration had no effect on body weight in rats maintained on HFD (*n* = 7–8/group). (B) Chronic treatment with Ex-Phe1 had no effect on HFD intake in rats (*n* = 7–8/group). (C) Ex-Phe1 chronic administration did not induce kaolin intake (a proxy for nausea in rodents). (D) Ex-Phe1 treatment had similar potency to Ex-4 at enhancing glycemic control during an OGTT in HFD rats (*n* = 7–8/group). (E) AUC analyses of blood glucose from 0 to 120 min during an OGTT. All data expressed as mean ± SEM. (F) Chronic, continuously-delivered Ex-Phe1 reduced body weight and noncumulative food intake (G) to a lesser magnitude and duration than Ex-4 in rats maintained on HFD (*n* = 5–8/group). (H) Similarly, Continuously-delivered Ex-Phe1 and Ex-4 induced kaolin intake for a shorter duration than Ex-4 after minipump implantation in rats maintained on HFD (*n* = 5–8/group). (I) Acute treatment with Ex-Phe1 induces a hyperglycemic response similar to Ex-4 during an OGTT in HFD rats (*n* = 12). (J) AUC analyses of blood glucose from 0 to 120 min during an OGTT. (A)–(I) Analysed with two-way repeated-measures ANOVA followed by Tukey’s post hoc test (*p* <0.05): *Vehicle versus Ex-4, #Ex-Phe1 versus Ex-4, @Vehicle versus Ex-Phe1. (E), (J) Analysed with one-way repeated-measures ANOVA followed by Tukey’s post hoc test. Means with different letters are significantly different (*p* <0.05).

Next, we sought to determine if the effects of Ex-Phe1 observed in mice are consistent in rats on HF diet. Additionally, to discern the effects of Ex-Phe1 on nausea/malaise, pica behaviour was assessed by kaolin intake, a validated proxy for nausea/malaise in rodents.^14^ As expected, chronic treatment with Ex-4 (3 μg/kg, b.i.d.) induced robust body weight loss and food intake suppression throughout the duration of treatment (Figure 3A,B). Rats treated with Ex-4 consumed significant quantities of kaolin throughout the duration of treatment (Figure 3C). Interestingly, chronic treatment with Ex-Phe1 (3 μg/kg, b.i.d.) had no effect on food intake or body weight and did not induce kaolin intake (Figure 3C). Ex-Phe1 is reported to improve glycemic control similarly to Ex-4 in lean mice and mice on HFHS diet.^12^ As such, we then performed an OGTT in rats following 12 days of treatment with Ex-4, Ex-Phe1, or vehicle. We observed improved glycemic control in rats treated with Ex-Phe1 relative to vehicle; however, Ex-Phe1 was less effective than Ex-4 (Figure 3D,E). We observed a similar pattern on body weight, food intake, and kaolin intake using a larger dose (7 μg/kg/day) delivered in an osmotic pump. Continuously delivered Ex-Phe1 initially reduced body weight and food intake relative to vehicle, but the effect on food intake occurred only within the 48 h after implantation, and reduction in body weight had disappeared by day 6 (Figure 3F,G). Compared to both vehicle-treated and Ex-Phe1 treatment, continuously delivered Ex-4 significantly decreased 48-hour food intake for 4 days and produced body weight loss that was sustained for the study duration. Interestingly, Ex-Phe1 and Ex-4 both increased kaolin intake relative to vehicle within the 48 h after implantation (Figure 3H). However, only Ex-4 significantly increased kaolin intake within days 2–4 and 4–6. These results imply that, in rats on HF diet, chronic biased agonism of the GLP-1R by Ex-Phe1 minimally maintains glycemic control; however, less effectively than Ex-4, and similarly Ex-Phe1 less effectively reduces body weight and food intake, with a shorter duration of nausea/malaise.

### 3.4 | Effects of Acute GLP-1R agonism versus biased agonism on glycemic control in rats

Rats chronically treated with Ex-4 demonstrated a lower baseline BG than vehicle or Ex-Phe1 (Figure 3D). To further understand the effects of biased agonism of the GLP-1R on glycemic control in rats, we performed an OGTT following acute treatment with vehicle, Ex-4, and Ex-Phe1 in a separate group of rats (*n* = 15). Interestingly, acute treatment with Ex-4 and Ex-Phe1 induced a hyperglycemic response relative to vehicle (Figure 3I,J). However, this response was slightly delayed in the Ex-Phe1 treated rats compared to Ex-4, which caused a significant spike in BG after only 20 min. These results suggest that acute biased agonism of the GLP-1R by Ex-Phe1 induces a hyperglycemic response similar to Ex-4 that is sustained.

### 3.5 | Effects of chronic GLP-1R agonism versus biased agonism in musk shrews

**FIGURE 4:**
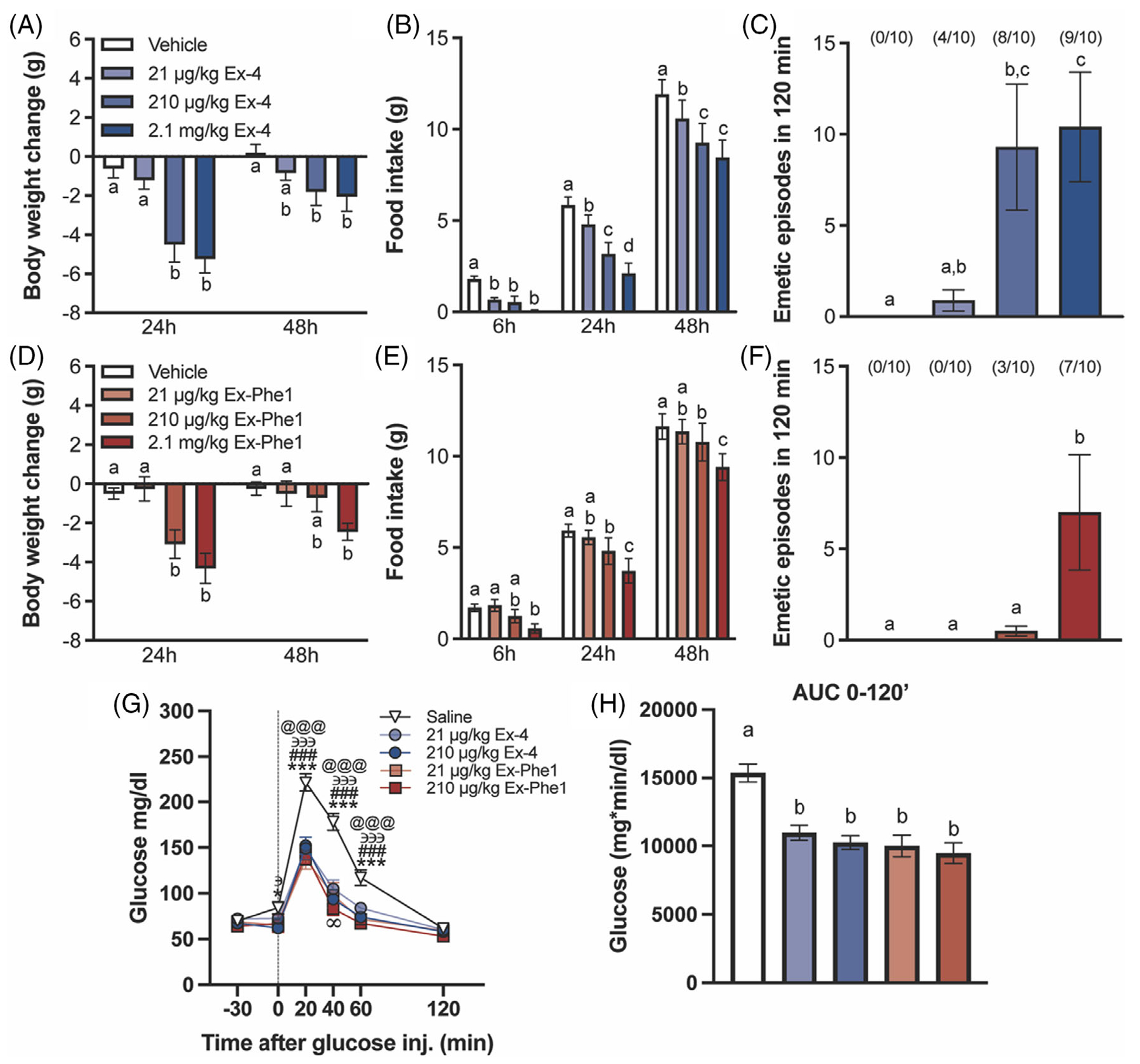
Effects of acute treatment with Ex-Phe1 in musk shrews. (A) Ex-4 dose-dependently reduces body weight and (B) suppresses food intake. (C) The number of single emetic episodes in 120 min following systemic administration of vehicle and various doses of Ex-4. (D) Ex-Phe1 modestly reduces body weight and (E) suppresses food intake at 210 μg/kg and 2.1 mg/kg, however to a lesser magnitude than Ex-4. (F) The number of single emetic episodes in 120 min following systemic administration of vehicle and various doses of Ex-Phe1. (G) Ex-Phe1 and Ex-4 show equal potency in enhancing glucose clearance during IPGTT (2 g/kg). (H) AUC analyses from 0 to 120 min. Ex-4 and Ex-Phe1 treated animals had lower AUC 0–120 compared to vehicle treated animals. For (A)–(E) *n* = 10, for (F), (G) *n* = 9). All data are expressed as mean ± SEM. In the emetic studies, the number of animals exhibiting emesis, expressed as a fraction of the total number of animals tested, is indicated above each treatment group. Data in (A)–(G) were analysed with two-way repeated-measures ANOVA followed by Tukey’s post hoc test. Data in (H) were analysed with one-way repeated measures ANOVA followed by Tukey’s post hoc test. Means with different letters are significantly different from each other (*p* <0.05). Vehicle versus 21 μg/kg Ex-Phe1: *p <0.05, ***p <0.0001; Vehicle versus 210 μg/kg Ex-Phe1: ###*p* <0.0001; Vehicle versus 21 μg/kg Ex-4: @@@*p* <0.0001; Vehicle versus 210 μg/kg Ex-4: ∋*p* <0.05, ∋∋∋*p* <0.001; 210 μg/kg Ex-Phe1 versus 21 μg/kg Ex-4: ∞*p* <0.05.

To further understand the impact of biased agonism of the GLP-1R by Ex-Phe1 on inducing nausea and emesis, we performed studies in an animal model capable of emesis using the musk shrew (*S. murinus*). The administration of Ex-4 (21, 210, and 2.1 mg/kg) induced a dose-dependent effect on weight loss (Figure 4A) and food intake (Figure 4B) reduction over 48 h. In addition, our results showed that Ex-4 also dose-dependently induced emesis (Figure 4C). Ex-Phe1 administration (21, 210, and 2.1 mg/kg) also induced significant reductions in body weight (Figure 4D) and food intake (Figure 4E) in a dose-dependent manner; however, it showed a lower emetic response compared to Ex-4, with a complete absence of emesis at the 21 μg/kg dose (Figure 4F). To evaluate the glycemic effects of Ex-Phe1 versus Ex-4, we conducted intraperitoneal glucose tolerance tests (IPGTT) in musk shrews. Ex-Phe1 treatment maintained glycemic control as effectively as Ex-4 (Figure 4G,H).

These findings indicate that acute administration of the GLP-1R-biased agonist, Ex-Phe1 in musk shrews significantly enhances glycemic control, reduces body weight and food intake, while producing substantially reduced nausea and emesis compared to the GLP-1R receptor agonist, Ex-4.

### 3.6 | Ex-Phe1 increases neuronal activation within the AP/NTS of mice, rats, and musk shrews

**FIGURE 5:**
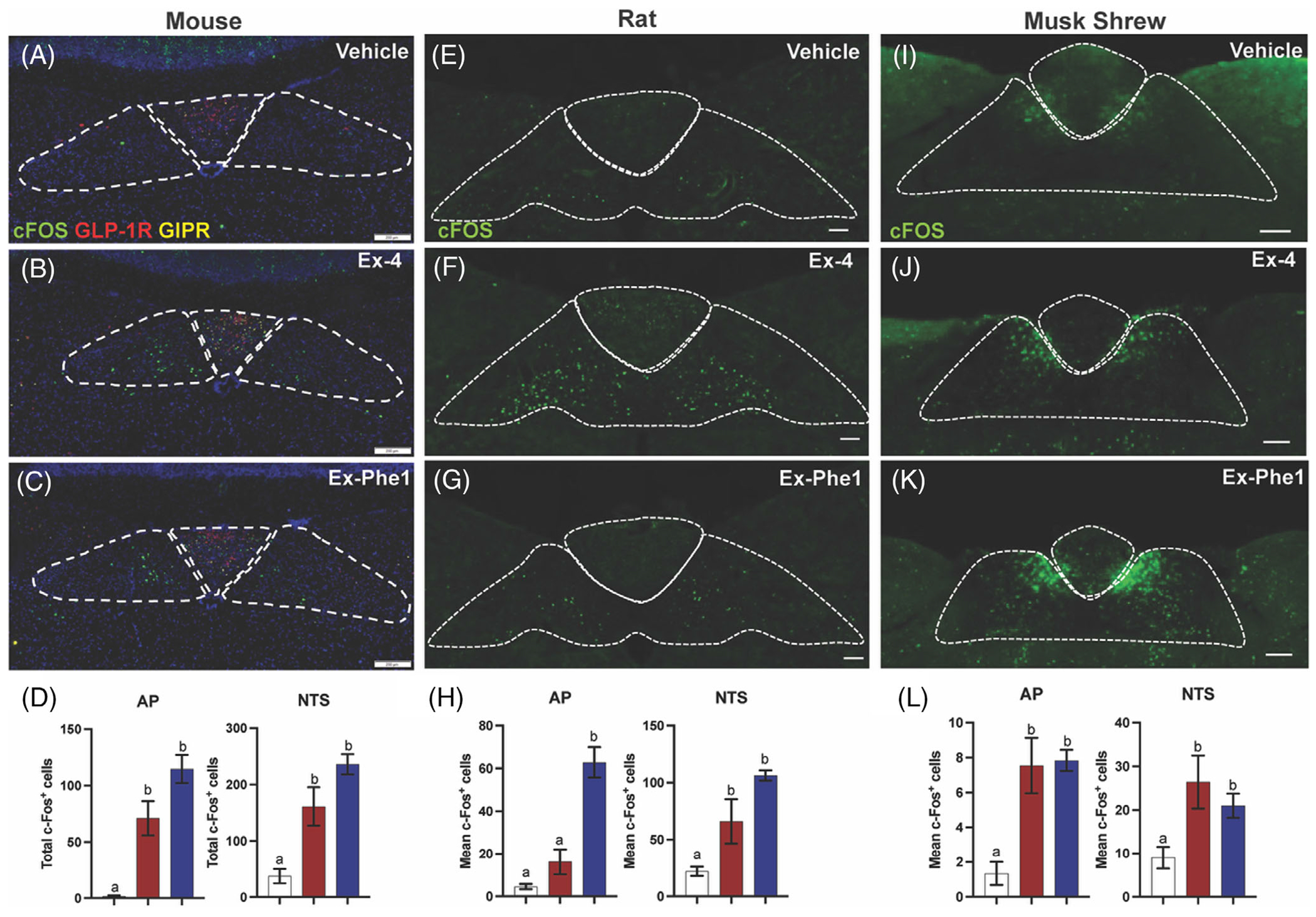
(A)–(C) Representative immunofluorescent images showing c-Fos positive cells in the area postrema (AP) and nucleus of the solitary tract (NTS) after vehicle (saline), Ex-4 (84 μg/kg, I.P.), or Ex-Phe1 (84 μg/kg, I.P.) in mice (*n* = 5/group). (D) Quantification of c-Fos-positive neurons in the AP and NTS of mice. (E)–(G) Representative immunofluorescent images showing c-Fos positive cells in the AP and NTS after vehicle (saline), Ex-4 (3 μg/kg, I.P.), or Ex-Phe1 (3 μg/kg, I.P.) in rats (*n* = 10/group). (I)–(K) Representative immunofluorescent images showing c-Fos positive cells in the AP and NTS after vehicle (saline), Ex-4 (210 μg/kg, I.P.), or Ex-Phe1 (210 μg/kg, I.P.) in shrews (*n* = 3–4/group). Data in (D)–(L) analysed using unpaired Studenťs *t*-tests. All data are expressed as mean ± SEM. Means with different letters are significantly different from each other (*p* <0.05). Images in (A)–(C) were acquired at 10× magnification. Images in (E)–(K) were acquired at 20× magnification. (A)–(C) Scale bar= 200 μm.

To elucidate the mechanism(s) underlying the effects of GLP-1R biased agonism by Ex-Phe1, we assessed c-*fos* or Fos expression within the AP and NTS of mice, rats, and musk shrews following treatment with Ex-4, Ex-Phe1, or vehicle. In all three species, Ex-Phe1 increased c-*fos*/Fos expression within the NTS relative to vehicle (Figure 5). c-*fos*/Fos expression within the NTS was not significantly different between Ex-4 and Ex-Phe1 treated animals of all three species (Figure 5D,H,L). In mice (Figure 5A–D) and musk shrews (Figure 5I–L), Ex-Phe1 also increased c-*fos*/Fos expression within the AP relative to vehicle and like Ex-4. Rats demonstrated increased c-*fos*/Fos expression within the AP relative to vehicle following Ex-Phe1; however, it was not significant but was significantly lower than c-*fos*/Fos expression observed within the AP of Ex-4 treated rats (Figure 5E–H).

## 4 | DISCUSSION

The effects of the GLP-1R biased agonist Ex-Phe1 were investigated in three different pre-clinical mammalian models (mice, rats, and musk shrews) to investigate the physiology of feeding and malaise behaviour. Compared to the widely used Ex-4, the anorectic effects of chronically administered Ex-Phe1 were more robust in mice and attenuated in musk shrews and rats. Nausea and emetic side effects were significantly reduced with Ex-Phe1 treatment compared to Ex-4 in rats, measured by kaolin intake, and musk shrews, measured by the number of emetic episodes. Interestingly, Ex-Phe1 enhances glycemic control similarly to Ex-4 in rats and musk shrews and has been reported to do so in mice.^12^ Collectively, these results highlight both the potential for GLP-1R biased agonists to retain therapeutic effects while minimising adverse side effects as well as the importance of investigating multiple preclinical species models to best evaluate the translational potential of this class of drugs.

The key difference between Ex-4 and Ex-Phe1 is that the latter shows biased signalling at the GLP-1R, with preserved cAMP signalling and reduced *β*arr relative to Ex-4 and GLP-1.^12^ The data presented here suggest *β*arr may play a role in mediating nausea/emesis associated with GLP-1R agonism; however, its effects remain unclear. Studies have shown *β*arr leads to receptor internalisation and may dampen the effects of GLP-1R agonism,^8,12,24^ but how this mediates nausea and emesis is not understood. As previously stated, biased agonists have been utilised for other GPCRs, such as the *μ*-opioid receptor. These biased agonists also demonstrate favoured G-protein signalling with limited *β*-arrestin recruitment and are associated with reduced nausea and vomiting compared to their unbiased counterparts.^9–11^ Despite our lack of understanding, clinical data from GLP-1R biased agonists ecnoglutide^25^ and MET-097^26^ reveal reduced prevalence of nausea and vomiting in patients. Hence, there is a need for further investigation to understand the role of *β*arr in mediating nausea/emesis which may have diverse pharmaceutical applications.

The species differences observed with the effects of biased agonism of GLP-1R by Ex-Phe1 are interesting. Multiple differences between species were observed in the present work, which highlights the need for caution in interpretation of data using a single preclinical model. In the present studies, differences may be generally explained by the different routes of administration (e.g., continuous vs. bolus), doses, dosing regimens (chronic vs. acute), or diets that the animals were maintained on throughout the study. While mice and rats were administered treatments chronically, this dosing regimen is not possible in the musk shrews, as they are extremely lean and cannot endure chronic energy deprivation due to relatively low body adipose stores in the species. Therefore, only acute dosing was conducted in the shrews. Doses for mice were determined based on the EC50 of Ex-Phe1 and administered via mini-pump to compare to published studies in mice testing a lower dose of Ex-Phe1.^12^ Rat doses are based on previous Ex-4 chronic administration studies that revealed 3 μg/kg b.i.d. causes robust kaolin consumption.^13^ The short duration of reduction in food intake seen in the osmotic mini-pump compared to peripheral administration aligns with other studies reporting a transient effect when anorexigenic substances are infused continuously, likely due to either compensatory mechanisms in anorexigenic and orexigenic signalling, or receptor downregulation and tolerance.^27^ However, in mice, the anorectic effects of Ex-4 and Ex-Phe1 were sustained throughout the duration of treatment, further highlighting species differences. Due to the high metabolic rate of musk shrews, they require higher dosing compared to mice and rats. Thus, doses for shrews were determined based on published studies that administered Ex-4 at 21 and 210 μg/kg and assessed glucose clearance, food intake, and emetic episodes.^28^ Here, the highest dose tested (2.1 mg/kg) was utilised to compare Ex-Phe1 to a dose of Ex-4 expected to cause significant malaise and emesis. Furthermore, mice and rats were maintained on a HFHS (40% fat, 20% sugar) or HF (60 kal% fat) diet, while the musk shrews were maintained on their standard feline (27.364% fat) diet. This difference in diets is due to the fact that musk shrews do not exhibit diet-induced obesity.

Aside from methodological differences, it is also possible that the species differences reported herein are due to differential renal clearances rates of the compounds, the role of β-arrestin, differences in tolerance, downstream signalling cascades, and/or differential GLP-1R expression in the CNS. However, we are unaware of any obvious mechanism(s) that contribute to these species differences besides the known differences with Ex-4 observed in the rat. Of relevance, species differences are observed between mice, rats, and musk shrews following acute administration of Ex-4. In rats, acute Ex-4 produces a hyperglycemic response^29^ that is absent in mice and musk shrews, suggesting there are differences in the central GLP-1R system between the species. We also observed this hyperglycemic response following acute treatment with Ex-Phe1 in rats, but not in mice^12^ or musk shrews. We do not yet know the effects of Ex-4 versus Ex-Phe1 on insulin secretion in rats or musk shrews, while studies in mice reflect sustained insulin secretion following Ex-Phe1 relative to Ex-4 and vehicle.^12^ Future studies will need to be conducted to investigate the effects on insulin secretion in rats and musk shrews. While other GLP-1R biased agonists have been investigated in pre-clinical models, the majority have been investigated in mice^12,30,31^ and very few have been tested in vivo in multiple species to assess the effects on food intake, body weight, and malaise/vomiting. For example, another similar GLP-1R biased agonist, ecnoglutide, suppresses food intake and body weight in both mice and rats,^25^ however the effects on malaise/vomiting in pre-clinical models is unknown. A multitude of future studies will be needed to fully investigate the mechanism(s) underlying these species differences and the similar and differential effects of all current and future GLP-1R biased agonists to better understand if targeting a GLP-1R biased agonist will translate to improved patient outcomes compared to existing GLP-1R pharmacotherapies.

The NTS and AP play a critical role in mediating the anorectic effects of GLP-1R agonism^32^ and in the control of nausea and eme-sis.^33^ Using *c-Fos* as a marker for neuronal activation, we observed increased *c-Fos* expression within the NTS of mice, rats, and musk shrews following Ex-4 or Ex-Phe1. However, the *c-Fos* expression within the NTS of mice and rats following Ex-Phe1 was slightly blunted relative to the expression following Ex-4. Published studies in mice suggest that the anorectic effects of GLP-1R agonism are mediated by the NTS, while the aversive effects are mediated by the AP.^34^ Interestingly, we observed differences in c-Fos expression within the AP of rats treated with Ex-Phe1 compared to Ex-4, while *c-Fos* expression within the AP of mice was slightly blunted, and musk shrews demonstrated similar *c-Fos* expression within the AP with Ex-Phe1 and Ex-4. While Ex-Phe1 led to food intake and body weight suppression in mice and musk shrews, this effect was absent in rats despite observing increased *c-Fos* expression within the NTS. Collectively, these results suggest that activation of both the AP and NTS is important for the food intake and body weight suppression associated with GLP-1R agonism. We did not extend our *c-Fos* expression analysis to other CNS nuclei associated with GLP-1R agonism and feeding regulation, such as the hypothalamus, hippocampus, or mesolimbic reward nuclei (see References [1,35] for review), as this work focused on the NTS and AP as sites known to mediate GLP-1R-induced suppression of food intake, body weight, as well as induce nausea and emesis.^32,34,36,37^ Future studies should investigate additional brain circuitry potentially associated with GLP-1R biased agonism.

GLP-1R agonists are highly effective for the treatment of obesity; however, in some clinical cases, such as patients with T2DM and comorbidities like cystic fibrosis, cancer, or HIV infection, the body weight and appetite loss characteristic of existing GLP-1R agonists is not advantageous. Thus, it becomes critically important to not only fully understand the mechanisms of these therapeutics to alleviate emesis, but also to determine whether selective metabolic outcomes of GLP-1 pharmacotherapy (e.g., glycemic control) can be achieved without weight loss and nausea/emesis through biased agonism. These results highlight the potential for designing GLP-1R agonists that preferentially activate cAMP components of the G-protein pathways, thereby minimising the adverse side effects (nausea and vomiting) mediated by *β*-arrestin and improving the therapeutic potential of GLP-1R based treatments.

## Supplementary Material

supplemental material

Additional supporting information can be found online in the Supporting Information section at the end of this article.

## Data Availability

The data that support the findings of this study are available from the corresponding author upon reasonable request.
