# Supplementary material for: GLP-1R biased cAMP agonism maintains glycemic control with reduced malaise and emesis in preclinical mammalian models
Source: Diabetes Obes Metab. Author manuscript; Available in PMC 2026 Mar 12. (PMC12981003; doi:10.1111/dom.70427)
Supplement: supplemental material [file NIHMS2146663-supplement-supplemental_material.docx]

**Supplemental Table 1**

*In vitro* pharmacology of agonists at species GLP-1R. Data are pharmacological parameters calculated from concentration-response data in Figure 1. EC_50_ values are reported as the geometric mean with the SEM and the number of observations in parentheses. *E*_max_ values are the arithmetic means ± SEM.

|  | cAMP EC_50_ (nM) | | cAMP E_max_ | | βarr-1 EC_50_ (nM) | | βarr-1 E_max_ | | βarr-2 EC_50_ (nM) | | βarr-2 E_max_ | |
| --- | --- | --- | --- | --- | --- | --- | --- | --- | --- | --- | --- | --- |
|  | GeoMean | Geo SEM | Mean | SEM | GeoMean | Geo SEM | Mean | SEM | GeoMean | Geo SEM | Mean | SEM |
| **human GLP1R** |  |  |  |  |  |  |  |  |  |  |  |  |
| GLP-1 (7-36) | 0.0182 | 0.0078 | 100.00 | 0.00 | 2.4062 | 0.5691 | 99.99 | 0.00 | 1.2322 | 0.2503 | 100.00 | 0.00 |
| Ex-4 | 0.0165 | 0.0058 | 95.92 | 1.82 | 1.5276 | 0.2686 | 96.44 | 0.56 | 1.0186 | 0.1703 | 96.05 | 1.29 |
| Ex-Phe1 | 0.1578 | 0.0146 | 101.84 | 4.08 | 4.8454 | 0.9078 | 34.34 | 1.85 | 3.7182 | 0.6200 | 45.47 | 2.00 |
| **rat GLP1R** |  |  |  |  |  |  |  |  |  |  |  |  |
| GLP-1 (7-36) | 0.0163 | 0.0044 | 100.00 | 0.00 | 1.2171 | 0.2938 | 99.99 | 0.00 | 0.9016 | 0.1675 | 100.00 | 0.00 |
| Ex-4 | 0.0146 | 0.0056 | 95.71 | 6.79 | 0.6829 | 0.1071 | 96.24 | 0.76 | 0.5641 | 0.0230 | 99.12 | 1.05 |
| Ex-Phe1 | 0.0386 | 0.0031 | 98.15 | 6.35 | 1.0225 | 0.1947 | 52.37 | 2.87 | 0.8961 | 0.2081 | 65.48 | 1.40 |
| **mouse GLP1R** |  |  |  |  |  |  |  |  |  |  |  |  |
| GLP-1 (7-36) | 0.0201 | 0.0089 | 100.00 | 0.00 | 1.2551 | 0.3696 | 100.00 | 0.00 | 1.5536 | 0.2610 | 100.00 | 0.00 |
| Ex-4 | 0.0176 | 0.0074 | 93.28 | 3.47 | 0.5649 | 0.1106 | 91.28 | 1.36 | 0.7345 | 0.1301 | 89.80 | 2.58 |
| Ex-Phe1 | 0.0414 | 0.0037 | 101.68 | 1.53 | 0.9506 | 0.2408 | 27.53 | 2.51 | 0.9727 | 0.2684 | 34.72 | 3.40 |
| **shrew GLP1R** |  |  |  |  |  |  |  |  |  |  |  |  |
| GLP-1 (7-36) | 0.0102 | 0.0060 | 100.00 | 0.00 | 1.7947 | 0.2717 | 99.99 | 0.01 | 1.8736 | 0.0998 | 100.00 | 0.00 |
| Ex-4 | 0.0043 | 0.0016 | 96.11 | 1.52 | 0.9085 | 0.1332 | 101.70 | 7.33 | 0.9354 | 0.1451 | 93.64 | 2.00 |
| Ex-Phe1 | 0.0253 | 0.0048 | 102.41 | 4.04 | 0.7709 | 0.0826 | 37.18 | 6.34 | 1.3709 | 0.0932 | 33.04 | 3.51 |
